# Maternal health and pregnancy outcomes among women of refugee background from African countries: a retrospective, observational study in Australia

**DOI:** 10.1186/s12884-014-0392-0

**Published:** 2014-11-27

**Authors:** Melanie Gibson-Helm, Helena Teede, Andrew Block, Michelle Knight, Christine East, Euan M Wallace, Jacqueline Boyle

**Affiliations:** Monash Centre for Health Research and Implementation, School of Public Health and Preventive Medicine, Monash University, Melbourne, Australia; Diabetes and Vascular Medicine Unit, Monash Health, Melbourne, Australia; Refugee Health Service, Monash Health, Melbourne, Australia; Monash Women’s Maternity Services, Monash Health, Melbourne, Australia; School of Nursing and Midwifery, Monash University, Melbourne, Australia; The Ritchie Centre, Monash University, Melbourne, Australia

**Keywords:** Refugee, Africa, Pregnancy, Birth, Pregnancy care, Maternal health, Migrant, Antenatal, Obstetric outcomes, Perinatal

## Abstract

**Background:**

Women of refugee background from Africa are reported to have a greater risk of adverse pregnancy outcomes compared to women born in resettlement countries. However, there is currently little insight into whether adverse pregnancy outcomes are more common among migrant women of refugee background, compared to women who have migrated for non-humanitarian reasons. To inform whether women of refugee background require additional services in pregnancy compared to non-refugee migrant women from similar world regions we aimed to describe and compare maternal health, pregnancy care attendance and pregnancy outcomes among migrant women from Africa with or without a refugee background.

**Methods:**

Retrospective, observational study of singleton births at a single, metropolitan, maternity service in Australia 2002–2011, to women born in humanitarian source countries (HSC) and non-HSC from North Africa (n = 1361), Middle and East Africa (n = 706) and West Africa (n = 106).

**Results:**

Compared to non-HSC groups, age < 20 years (0–1.4% vs 2.3-13.3%), living in relatively socio-economically disadvantaged geographic areas (26.2-37.3% vs 52.9-77.8%) and interpreter need (0–23.9% vs 9.7-51.5%) were generally more common in the HSC groups. Compared to non-HSC groups, female genital mutilation (0.3-3.3% vs 5.1-13.8%), vitamin D insufficiency (8.7-21.5% vs 23.3-32.0%), syphilis (0–0.3% vs 1.2-7.5%) and hepatitis B (0–1.1% vs 1.2-18%) were also generally more common among the HSC groups. Unplanned birth before arrival at the hospital (3.6%) was particularly high in the North African HSC group. HSC-birth was associated with gestational diabetes mellitus (odds ratio = 3.5, 95% confidence interval: 1.8-7.1) among women from Middle and East Africa, after adjusting for maternal age, parity, body mass index and relative socio-economic disadvantage of area of residence. The West African HSC group had the highest stillbirth incidence (4.4%).

**Conclusions:**

Migrant women of refugee background from different African regions appear to be at greater risk of specific adverse pregnancy outcomes compared to migrant women without a refugee background. Awareness of differing risks and health needs would assist provision of appropriate pregnancy care to improve the health of African women and their babies.

## Background

For the last decade Africa has been a key focus of humanitarian migration programs internationally [1,2]. Compared to women born in resettlement countries, women of refugee background in general [3,4], and women from African humanitarian source countries specifically [5,6], are reported to have greater risks of adverse pregnancy outcomes such as stillbirth [3,6], perinatal mortality [3-5] and caesarean section [6]. A number of studies have also reported greater risks of adverse pregnancy outcomes among *migrant women in general* from African countries, compared to women born in resettlement countries including stillbirth [7,8], perinatal mortality [9-11], caesarean section [11,12], preterm birth [10,12] and low birth weight [11,12]. Previous research has concluded that the relationship between migration and pregnancy outcomes varies with migrant subgroup (defined by race/ethnicity or world region of origin), and resettlement country [13]; however, there is currently little insight into the impact of refugee background over and above that of migrant background among women from Africa. Comparing pregnancy outcomes between migrant women of refugee and non-refugee background from similar world regions enables exploration of whether women of refugee background are at additional risk of adverse outcomes and require pregnancy services different to those for women who have migrated for non-humanitarian reasons.

Interpreting pregnancy outcomes for a heterogeneous migrant population may be limited by possible bias due to differences between women that are often undetectable in routine clinical datasets (e.g. cultural beliefs, dietary habits or genetic background). For example, female genital mutilation (FGM) is a risk factor for adverse pregnancy outcomes that varies in prevalence and severity between African regions [14]. Investigating maternal health and pregnancy outcomes in regional subgroups of women may identify needs specific to particular groups of resettled refugees.

Accordingly, we aimed to report maternal health, pregnancy care attendance and pregnancy outcomes among three cohorts of women born in African humanitarian source countries (HSC) compared to women born in African non-HSC, at one of Australia’s largest health services.

## Methods

Women of refugee background may come to Australia after being granted a visa overseas through the Humanitarian Migration Program, by boat or plane and applying for asylum after arrival, or through the Family or Skilled Migration Programs (having gained a non-humanitarian visa but being of refugee background) [15]. Generally, between 10,000 and 15,000 people per year have arrived in Australia through the Humanitarian Program since 1985 [1]; the four countries of birth that contributed the most arrivals through the Humanitarian Program between 2002 and 2011 were Sudan, Afghanistan, Iraq and Burma [16]. The local government areas of Greater Dandenong and Casey (served by Monash Health) continue to be major areas of refugee resettlement receiving more than 25% of Victoria’s approximately 14,200 Humanitarian Program arrivals in 2010–2013 [15].

Monash Health is the largest public health service and the largest maternity provider in Victoria, providing maternity care across three hospitals in Melbourne’s south-east. Between 2002 and 2011 there were approximately 6,500 births each year at Monash Health and half of the women giving birth there were born overseas.

Routinely collected data from clinical care can make valuable contributions to medical research, but the selection of data fields for collection impacts on the options available to researchers [17]. In this study dataset, country of birth was the only routinely collected data field that could act as an indicator of possible refugee background. Taking this into account, we used national immigration data [16] for 2002–2011 to identify African countries of birth from where two thirds or more of the total immigrants had entered Australia within the humanitarian stream (HSC) and countries of birth from where only one third or fewer had entered within the humanitarian stream (non-HSC).

All women receiving free (government funded), universal healthcare with a singleton pregnancy and giving birth at Monash Health between 2002 and 2011 were selected if their country of birth was in the African HSC category. From each United Nations-defined African region [18] represented in the HSC group, all countries of birth in the non-HSC category were also selected from the Monash Health data to form a comparison (control) group.

Data were extracted from the Birthing Outcomes System, an electronic database recording all births (liveborn or stillborn) of ≥20 weeks gestation. Data are routinely entered into the Birthing Outcomes System by midwifery staff during the first pregnancy care visit at the hospital and at birth, with routine data maintenance, cleaning and validation. This study was deemed a quality assurance project (12110Q) by the Monash Health Human Research Ethics Committee Medical Administrator.

Postcodes of residence were matched to the corresponding Australian Bureau of Statistics Index of Relative Socio-economic Disadvantage (IRSD) decile [19]: decile 1 = relatively most socio-economically disadvantaged geographic areas, decile 10 = relatively least disadvantaged. Categories consistent with Australia’s annual perinatal reports [20] were used for maternal age, late first pregnancy care visit at the hospital (≥14 weeks gestation), pre-term birth (<37 weeks gestation), post-term birth (>41 weeks gestation), low birth weight (<2500 g) and low five-minute Apgar score (<7). Poor attendance was defined as two or more booked pregnancy care visits at the hospital missed. Cephalic presentation included vertex, face and brow; non-cephalic included breech, shoulder, cord, compound and other (unknown presentation excluded). The denominator for previous caesarean sections was multiparous women. Onset of labour included spontaneous and induced (no labour excluded). Analgesia use during labour was analysed for women with spontaneous or induced labour. Assisted vaginal births, episiotomies and 3^rd^/4^th^ degree tears were analysed for vaginal births. Birth weight, low birth weight, five-minute Apgar score, admission to special or intensive neonatal care units, and neonatal length of stay were analysed for live births. Mother requiring high dependency care referred to admission to a specialist unit such as intensive care or coronary care, or a prolonged stay/return to the birth suite due to post-birth complications. Foetal abnormality was a condition in the foetus affecting the care or surveillance of the pregnancy e.g. a structural or anatomical abnormality. Interpreter requirement was collected from 2008; first pregnancy care visit at the hospital and analgesia use during labour were collected from 2009. Body mass index (BMI) was collected from 2005 but consistently from 2008. Routine vitamin D insufficiency/deficiency screening was introduced in 2009 (25-OH vitamin D serum level < 75 nmol/L). Singleton and multiple pregnancies have different risk profiles for pregnancy outcomes and there were not enough multiple pregnancies in each region (82 in total) to investigate pregnancy outcomes for singleton and multiple pregnancies separately and so multiple pregnancies were excluded.

Statistical analysis was performed with Stata software version 12.1 (StataCorp, College Station, Texas, USA). Analysis methodology and results were reviewed by an experienced biostatistician. Categorical data are presented as count and proportions and groups compared using Pearson chi-square tests or Fisher exact tests. Continuous data are presented as mean ± standard deviation (SD) or median and interquartile range (IQR), and groups compared using t-tests or Mann–Whitney U tests. A two-sided p-value of 0.05 was considered statistically significant.

Univariable logistic or linear regression analysis was conducted to generate crude odds ratios or β-coefficients, and 95% confidence intervals (CI), for associations between maternal HSC-birth and pregnancy outcomes. Non-symmetrically distributed outcomes were log-transformed for regression analysis. Multivariable logistic or linear regression analysis then assessed whether associations between HSC-birth and pregnancy outcomes were independent of four potential confounders: maternal age [<20 years old, 20–34 years (reference category), 35 years or older], parity [0 previous births (primiparity), ≥1 previous births (multiparity)], BMI [<18.5, 18.5-24.99 (reference category), 25.00-max] and IRSD [deciles 1–3 (relatively most socioeconomically disadvantaged geographic areas), deciles 4–10]. Complete data for age, parity, BMI and IRSD were available for 961 women from North Africa, 491 from Middle and East Africa and 82 from West Africa. Women who were missing data regarding BMI and/or IRSD were excluded from any multivariable analysis that included these variables. Categories with low numbers were collapsed as appropriate. Fewer confounders were included if number of cases was low to avoid over-fitting.

## Results

General health and demographic characteristics of the study populations are summarised in Table 1.

**Table 1:** **Demographics characteristics of women born in African non-humanitarian and humanitarian source countries**

	**North Africa**			**Middle and East Africa**			**West Africa**		
**Maternal health and wellbeing**	**Non-HSC group (%)**	**HSC group (%)**	**χ** ^**2**^ **p-value**	**Non-HSC group (%)**	**HSC group (%)**	**χ** ^**2**^ **p-value**	**Non-HSC group (%)**	**HSC group (%)**	**χ** ^**2**^ **p-value**
Age <20 years	3 (1.4)	77 (6.7)	<0.01	6 (1.0)	2 (2.3)	0.26	0	6 (13.3)	-
Age ≥35 years	42 (19.6)	190 (16.6)	0.27	138 (22.3)	23 (26.4)	0.39	21 (34.4)	2 (4.4)	<0.01
≥1 previous birth at ≥20 weeks gestation (multiparous)	133 (62.2)	919 (80.1)	<0.01	349 (56.4)	61 (70.1)	0.02	41 (67.2)	31 (68.9)	0.86
BMI < 18.5 (>2007, 30 missing data)	1/90 (1.1)	33/529 (6.2)	0.05	12/309 (3.9)	2/37 (5.4)	0.65	1/30 (3.3)	0/31	-
BMI ≥ 25.0 (>2007, 30 missing data)	68/90 (75.6)	232/529 (43.9)	<0.01	177/309 (57.3)	19/37 (51.4)	0.49	19/30 (63.3)	19/31 (61.3)	0.87
High relative socio-economic disadvantage (IRSD) (10 missing data)	73/212 (34.4)	762/1147 (66.4)	<0.01	228/611 (37.3)	46/87 (52.9)	<0.01	16 (26.2)	35 (77.8)	<0.01
Interpreter required (>2007, 34 missing data)	21/88 (23.9)	273/530 (51.5)	<0.01	2/305 (0.7)	15/37 (40.5)	<0.01	0	3/31 (9.7)	-
Married/de facto (174 missing data)	182/187 (97.3)	916/1101 (83.2)	<0.01	484/532 (91.0)	62/79 (78.5)	<0.01	55/57 (96.5)	29/43 (67.4)	<0.01
Female genital mutilation (FGM)	1 (0.5)	58 (5.1)	<0.01	2 (0.3)	12 (13.8)	<0.01	2 (3.3)	3 (6.7)	0.65
Previous caesarean section (multiparous only)	39/133 (29.3)	139/919 (15.1)	<0.01	97/349 (27.8)	15/61 (24.6)	0.60	13/41 (31.7)	4/31 (12.9)	0.06
Anemia	12 (5.6)	88 (7.7)	0.29	30 (4.9)	4 (4.6)	1.00	8 (13.1)	0	-
Vitamin D deficiency (>2008)	14/65 (21.5)	110/404 (27.2)	0.33	54/619 (8.7)	7/30 (23.3)	0.01	7/61 (11.5)	8/25 (32.0)	0.02
Syphilis	0	86 (7.5)	-	2 (0.3)	1 (1.2)	0.33	0	1 (2.2)	-
Hepatitis B	0	90 (7.9)	-	7 (1.1)	1 (1.2)	1.00	0	8 (17.8)	-
Hepatitis C	0	23 (2.0)	-	1 (0.2)	0	-	0	0	-
Human Immunodeficiency Virus	0	3 (0.3)	-	2 (0.3)	0	-	0	0	-

Data are presented as count (proportions). HSC: humanitarian source countries; BMI: body mass index; IRSD: Index of Relative Socio-economic Disadvantage.

Except where specified, North African non-HSC group n = 214, North African HSC group n = 1147, Middle and East African non-HSC group n = 619, Middle and East African HSC group n = 87, West African non-HSC group n = 61 and West African HSC group n = 45.

### North Africa

North African non-HSC included Algeria, Egypt, Libya, Morocco and Tunisia (n = 214); the only HSC was Sudan (n = 1147).

Compared to the North African non-HSC group, mean age was lower in the North African HSC group (39.5 ± 5.3 vs 28.1 ± 6.0 years, p < 0.01) and a larger proportion of the HSC group were aged <20 years (Table 1). Compared to the non-HSC group, the median BMI (27.0 (5.0) vs 24.0 (6.0) years, p < 0.01) was lower in the HSC group, a larger proportion of the HSC group had a BMI < 18.5 and fewer had a BMI ≥ 25.0. Larger proportions of the HSC group were multiparous, lived in a relatively socio-economically disadvantaged area, required an interpreter or had female genital mutilation (FGM). A smaller proportion of the HSC group reported a spouse and fewer multiparous women reported a previous caesarean section. There were no cases of syphilis, hepatitis B, hepatitis C or Human Immunodeficiency Virus (HIV) in the non-HSC group.

Table 2 summarises pregnancy care attendance and pregnancy outcomes of the women born in North Africa. There were no differences in adequacy of pregnancy care attendance and the majority of women in both groups had their first pregnancy care visit after the first trimester. There were no cases of unplanned birth before arrival at the hospital (BBA) in the non-HSC group and 41 cases in the HSC group. A smaller proportion of the North African HSC group developed gestational diabetes mellitus (GDM), had non-cephalic presentation, induced labour, analgesia use in labour, caesarean section, assisted vaginal birth, or episiotomy. Of the women having caesarean sections, 52.2% of the non-HSC group (36/69) and 75.1% of the HSC group (136/181) had emergency caesarean sections (p < 0.01). Of the multiparous women having caesarean sections, 77.3% of the non-HSC group (34/44) and 51.6% of the HSC group (65/126) had previously had a caesarean section (p < 0.01). Median maternal and neonatal lengths of stay were both shorter in the HSC group (Table 2). When adjusted for potential confounders such as age, parity, BMI and IRSD, the HSC group was less likely to experience non-cephalic presentation, caesarean section or assisted vaginal birth (Table 2).

**Table 2:** **Pregnancy care attendance and outcomes among women from North African non-HSC and HSC**

**Attendance and outcomes (categorical)**	**Non-HSC (%)**	**HSC (%)**	**χ** ^**2**^ **p-value**	**Crude odds ratio (95% CI)**	**Adjusted odds ratio (95% CI)**
Late first pregnancy care visit (>2008, 30 missing)	36/65 (55.4)	244/374 (65.2)	0.13	1.5 (0.9 to 2.6) n = 439	1.5 (0.8 to 2.6) n = 435
Poor/no pregnancy care attendance	5 (2.3)	58 (5.1)	0.08	2.2 (0.9 to 5.6)	2.9 (0.7 to 12.6)
Gestational diabetes mellitus	25 (11.7)	46 (4.0)	<0.01	0.3 (0.2 to 0.5)	0.5 (0.3 to 1.2)
Pre-eclampsia	3 (1.4)	23 (2.0)	0.79	1.4 (0.4 to 4.8)	1.4^a^ (0.4 to 4.6) n = 1361
Antepartum haemorrhage	6 (2.8)	16 (1.4)	0.14	0.5 (0.2 to 1.3)	0.5^b^ (0.2 to 1.3) n = 1361
Unplanned birth before hospital arrival	0	41 (3.6)			
Non-cephalic presentation (ex 6 unknown)	12/214 (5.6)	27/1141 (2.4)	0.01	0.4 (0.2 to 0.8) n = 1355	0.4^c^ (0.2 to 0.8) n = 1355
Induced labour (ex 96 no labour)	46/176 (26.1)	181/1089 (16.6)	<0.01	0.6 (0.4 to 0.8) n = 1265	0.8 (0.5 to 1.4) n = 892
Analgesia in labour (>2008, ex no labour, 1 missing)	34/45 (75.6)	178/383 (46.5)	<0.01	0.3 (0.1 to 0.6) n = 428	0.4 (0.2 to 1.0) n = 425
Caesarean section	69 (32.2)	181 (15.8)	<0.01	0.4 (0.3 to 0.5)	0.4 (0.3 to 0.7)
Assisted vaginal birth (vaginal births)	32/145 (22.1)	63/966 (6.5)	<0.01	0.2 (0.1 to 0.4) n = 1111	0.4 (0.2 to 0.8) n = 780
Episiotomy (vaginal births)	32/145 (22.1)	141/966 (14.6)	0.02	0.6 (0.4 to 0.9) n = 1111	1.0 (0.5 to 1.8) n = 780
3rd/4th degree tears (vaginal births)	2/145 (1.4)	26/966 (2.7)	0.57	2.0 (0.5 to 8.4) n = 1111	2.4^b^ (0.6 to 10.6) n = 1111
Postpartum haemorrhage	2 (0.9)	3 (0.3)	0.18		
Mother requiring high dependency care (6 missing)	0/214	13/1141 (1.1)			
Preterm birth	8 (3.7)	58 (5.1)	0.41	1.4 (0.6 to 2.9)	1.0 (0.3 to 2.8)
Post-term birth	4 (1.9)	53 (4.6)	0.07	2.5 (0.9 to 7.1)	2.0^d^ (0.7 to 5.8) n = 1359
Foetal abnormality	2 (0.9)	14 (1.2)	1.00	1.3 (0.3 to 5.8)	
Stillbirth	3 (1.4)	18 (1.6)	1.00	1.1 (0.3 to 3.8)	0.9^a^ (0.3 to 3.2) n = 1361
Low birth weight^e^	4/211 (1.9)	42/1129 (3.7)	0.18	2.0 (0.7 to 5.6) n = 1340	2.6^d^ (0.8 to 8.7) n = 1338
Apgar score <7^e^ (5 min) (10 missing)	2/211 (1.0)	21/1119 (1.9)	0.56	2.0 (0.5 to 8.6) n = 1330	1.9^a^ (0.4 to 8.4) n = 1330
Admission to special or intensive neonatal care (3 missing)	35/210 (16.7)	149/1127 (13.2)	0.18	0.8 (0.5 to 1.1) n = 1337	0.8 (0.5 to 1.3) n = 947
**Pregnancy outcomes (continuous)**	**Non-HSC**	**HSC**	**p-value**	**Crude β co-efficient (95%CI)**	**Adjusted β co-efficient (95% CI)**
Median (IQR) maternal length of stay (days)	3.1 (1.9)	2.4 (1.7)	<0.01	−0.14 (−0.23 to −0.06)^f^	−0.06 (−0.16 to 0.04)^f^
Median (IQR) neonatal length of stay^e^ (days) (2 missing)	2.8 (2.1) n = 210	2.2 (1.6) n = 1128	<0.01	−0.15 (−0.26 to −0.05)^f^ n = 1338	−0.09 (−0.22 to 0.04)^f^ n = 948
Median (IQR) gestation at birth (weeks)	39.6 (1.9)	39.6 (1.9)	0.57	0.00 (−0.01 to 0.01)^f^	0.01 (−0.01 to 0.02)^f^
Mean ± SD birth weight^e^ (grams)	3371.2 (±465.9) n = 211	3302.0 (±484.9) n = 1129	0.06	−69.23 (−140.15 to 1.67) n = 1340	26.30 (−62.37 to 114.96) n = 949

HSC: humanitarian source countries, CI: confidence interval, IQR: interquartile range, SD: standard deviation.

Odds ratios and β co-efficients were adjusted for age, parity, IRSD and BMI, except ^a^= adjusted for age, ^b^= adjusted for parity, ^c^= age, parity, ^d^= age, parity, IRSD.

Except where specified, n = 214 for the non-HSC group and 1147 for the HSC group for chi-squared tests, n = 1361 for univariable regression analysis and n = 961 for multivariable regression analysis.

^e^Live births only.

^f^Outcome log transformed for regression analysis.

### Middle and East Africa

Non-HSC included Angola, Comoros, Kenya, Mauritius, Mozambique, Seychelles, Zambia and Zimbabwe (n = 619); HSC included Democratic Republic of the Congo, Burundi, Eritrea, Rwanda and Tanzania (n = 87).

Compared to the Middle and East African non-HSC group, mean age was greater in the Middle and East African HSC group (30.3 ± 5.3 vs 31.9 ± 5.1 years, p = 0.01) but there was no significant difference in median BMI (25.0 (7.0) vs 25.0 (7.0), p = 0.54). Compared to the non-HSC group, larger proportions of the HSC group were multiparous, lived in a relatively socio-economically disadvantaged area, required an interpreter, or had FGM (Table 1). A smaller proportion of the HSC group reported having a spouse. Vitamin D deficiency/insufficiency was more common in the HSC group. There were no cases of hepatitis C or HIV in the HSC group.

Table 3 summarises pregnancy care attendance and pregnancy outcomes of the women born in Middle and East Africa. Half of the women in both groups had their first pregnancy care visit at the hospital after the first trimester. Compared to the non-HSC group, a larger proportion of the Middle and East African HSC group developed GDM. A smaller proportion of women born in Middle and East African HSC used analgesia in labour. There was no difference in emergency caesarean sections (non-HSC: 112/182, 61.5% vs HSC: 18/28, 64.3% p = 0.78) or repeat caesarean sections (non-HSC: 75/99, 75.8% vs HSC: 12/17, 70.6% p = 0.76). Median neonatal length of stay was longer in the HSC group. When adjusted for maternal age, parity, BMI and IRSD, the HSC group was more likely to experience GDM and less likely to use analgesia.

**Table 3:** **Pregnancy care attendance and outcomes among women from Middle and East African non-HSC and HSC**

**Attendance and outcomes (categorical)**	**Non-HSC (%)**	**HSC (%)**	**χ** ^**2**^ **p-value**	**Crude odds ratio (95% CI)**	**Adjusted odds ratio (95% CI)**
Late first pregnancy care visit (>2008, 8 missing)	124/234 (53.0)	15/29 (51.7)	0.90	0.9 (0.4 to 2.1) n = 263	0.9 (0.4 to 2.0) n = 261
Poor/no pregnancy care attendance	9 (1.5)	4 (4.6)	0.06	3.3 (1.0 to 10.8)	
Gestational diabetes mellitus (ex 1 case pre-existing diabetes)	49/618 (7.9)	19/87 (21.8)	<0.01	3.2 (1.8 to 5.8) n = 705	3.5 (1.8 to 7.1) n = 490
Pre-eclampsia	13 (2.1)	2 (2.3)	0.71	1.1 (0.2 to 4.9)	
Antepartum haemorrhage	21 (3.4)	2 (2.3)	1.00	0.7 (0.1 to 2.9)	0.7^b^ (0.2 to 3.0) n = 706
Unplanned birth before hospital arrival	6 (1.0)	1 (1.2)	0.60		
Non-cephalic presentation (ex 1 unknown)	26/619 (4.2)	2/86 (2.3)	0.56	0.5 (0.1 to 2.3) n = 705	0.5^b^ (0.1 to 2.4) n = 705
Induced labour (ex 103 no labour)	134/527 (25.4)	19/76 (25.0)	0.94	1.0 (0.6 to 1.7) n = 603	1.0 (0.5 to 2.0) n = 417
Analgesia in labour (>2008, ex no labour)	154/211 (73.0)	11/27 (40.7)	<0.01	0.2 (0.1 to 0.6) n = 238	0.3 (0.1 to 0.7) n = 235
Caesarean section	182 (29.4)	28 (32.2)	0.59	1.1 (0.7 to 1.8)	0.8 (0.4 to 1.6)
Assisted vaginal birth (vaginal births)	67/437 (15.3)	7/59 (11.9)	0.48	0.7 (0.3 to 1.7) n = 496	1.0 (0.3 to 2.8) n = 342
Episiotomy (vaginal births)	75/437 (17.2)	13/59 (22.0)	0.36	1.4 (0.7 to 2.6) n = 496	1.4 (0.6 to 3.2) n = 342
3rd/4th degree tears (vaginal births)	16/437 (3.7)	2/59 (3.4)	1.00	0.9 (0.2 to 4.1) n = 496	
Postpartum haemorrhage	18 (2.9)	1 (1.2)	0.49	0.4 (0.1 to 2.9)	
Mother requiring high dependency care	5 (0.8)	1 (1.2)	0.55		
Preterm birth	45 (7.3)	8 (9.2)	0.52	1.3 (0.6 to 2.8)	1.1 (0.4 to 3.4)
Post-term birth	9 (1.5)	1 (1.2)	1.00	0.8 (0.1 to 6.3)	
Foetal abnormality	7 (1.1)	2 (2.3)	0.31		
Stillbirth	4 (0.7)	1 (1.2)	0.48		
Low birth weight^e^ (1 missing data point)	33/614 (5.4)	3/86 (3.5)	0.61	0.6 (0.2 to 2.1) n = 700	0.6^c^ (0.2 to 2.1) n = 700
Apgar score <7^e^ (5 min) (1 missing)	13/614 (2.1)	2/86 (2.3)	0.71	1.1 (0.2 to 5.0) n = 700	
Admission to special or intensive neonatal care units^e^ (2 missing)	110/614 (17.9)	20/85 (23.5)	0.21	1.4 (0.8 to 2.4) n = 699	1.1 (0.5 to 2.2) n = 488
**Pregnancy outcomes (continuous)**	**Non-HSC**	**HSC**	**p-value**	**Crude β co-efficient (95% CI)**	**Adjusted β co-efficient (95% CI)**
Median (IQR) maternal length of stay (days)	2.8 (2.0)	3.2 (2.2)	0.15	0.04 (−0.08 to 0.16)^f^	−0.04 (−0.18 to 0.10)^f^
Median (IQR) neonatal length of stay^e^ (days)	2.5 (2.0) n = 615	3.4 (2.1) n = 86	0.01	0.18 (0.00 to 0.36)^f^ n = 701	0.05 (−0.16 to 0.26)^f^ n = 488
Median (IQR) gestation at birth	39.5 (1.8)	39.5 (2.4)	0.54	−0.01 (−0.02 to 0.01)^f^	0.01 (−0.01 to 0.03)^f^
Mean ± SD birth weight^e^ (grams) (1 missing)	3294.1 ± 574.3 n = 614	3307.6 ± 532.6 n = 86	0.84	13.49 (−115.23 to 142.21) n = 700	9.15 (−142.19 to 160.48) n = 487

HSC: humanitarian source countries, CI: confidence interval, IQR: interquartile range, SD: standard deviation.

Odds ratios and β co-efficients were adjusted for age, parity, IRSD and BMI, except ^b^= adjusted for parity, ^c^= age, parity.

Except where specified, n = 619 for the non-HSC group and 87 for the HSC group for chi-squared tests, n = 706 for univariable regression analysis and n = 491 for multivariable regression analysis.

^e^Live births only.

^f^Outcome log transformed for regression analysis.

### West Africa

West African non-HSC included Burkina Faso, Ghana, Mali and Nigeria (n = 61); HSC included Guinea, Liberia, Mauritania and Sierra Leone (n = 45).

Compared to the non-HSC group, women born in HSC in West Africa had a lower mean age (32.2 ± 5.2 vs 25.5 ± 5.2 years, p < 0.01), a smaller proportion were 35 years or older and a larger proportion were aged less than 20 years (Table 1). There was no significant difference in median BMI (27.0 (8.0) vs 26.0 (6.0), p = 0.45). Compared to the non-HSC group, a larger proportion of the HSC group lived in a relatively socio-economically disadvantaged area. No women required an interpreter in the non-HSC group. A smaller proportion of the HSC group reported a spouse and a larger proportion had vitamin D deficiency/insufficiency. There were no cases of syphilis or hepatitis B in the non-HSC group and no cases of hepatitis C or HIV in either group.

Table 4 summarises pregnancy care attendance and pregnancy outcomes of the women born in West Africa. Compared to the non-HSC group, fewer women in the West African HSC group had caesarean sections however there was no difference in emergency caesarean sections (non-HSC: 11/26, 42.3% vs HSC: 8/10, 80.0% p = 0.07) or repeat caesarean sections (non-HSC: 12/17, 70.6% vs HSC: 4/7, 57.1% p = 0.65). Due to the small West African population size, regression analysis was conducted only for continuous outcomes. Length of stay and gestation at birth were non-symmetrically distributed and therefore log-transformed. After adjusting for maternal age, parity, BMI and IRSD there were no independent associations between maternal HSC-birth and maternal length of stay (β-coefficient = −0.19, 95% CI: −0.52 to 0.14), neonatal length of stay (β-coefficient = −0.27, 95% CI: −0.66 to 0.12), gestation at birth (β-coefficient = −0.01, 95% CI: −0.03 to 0.01) or birth weight (β-coefficient = −247.02, 95% CI: −503.78 to 9.74).

**Table 4:** **Pregnancy care attendance and outcomes among women from West African non-HSC and HSC**

**Attendance and outcomes (categorical)**	**Non-HSC group (%)**	**HSC group (%)**	**χ** ^**2**^ **p-value**
Late first pregnancy care visit (>2008, 3 missing data)	17/22 (77.3)	19/24 (79.2)	1.00
Poor/no pregnancy care attendance	3 (4.9)	1 (2.2)	0.64
Gestational diabetes mellitus	2 (3.3)	1 (2.2)	1.00
Pre-eclampsia	3 (4.9)	0	
Antepartum haemorrhage	6 (9.8)	0	
Unplanned birth before hospital arrival	1 (1.6)	0	
Non-cephalic presentation	5 (8.3)	1 (2.2)	0.23
Induced labour (ex 22 cases of no labour)	7/42 (16.7)	6/42 (14.3)	0.76
Analgesia in labour (>2008, ex no labour)	12/14 (85.7)	17/24 (70.8)	0.44
Caesarean section	26 (42.6)	10 (22.2)	0.03
Assisted vaginal birth (vaginal births)	2/35 (5.7)	2/35 (5.7)	1.00
Episiotomy (vaginal births)	4/35 (11.4)	5/35 (14.3)	1.00
3rd/4th degree tears (vaginal births)	1/35 (2.9)	1/35 (2.9)	1.00
Postpartum haemorrhage	2 (3.3)	0	
Preterm birth	8 (13.1)	3 (6.7)	0.35
Foetal abnormality	1 (1.6)	0	
Stillbirth	1 (1.6)	2 (4.4)	0.57
Low birth weight^e^	6/60 (10.0)	0/43	
Apgar score <7^e^ (5 min)	2/60 (3.3)	0/43	
Admission to special or intensive neonatal care units^e^	13/60 (21.7)	5/43 (11.6)	0.19
**Pregnancy outcomes (continuous)**	**Non-HSC**	**HSC**	**p-value**
Median (IQ) maternal length of stay (days)	2.9 (2.0)	2.2 (2.1)	0.07
Median (IQR) neonatal length of stay (days)	2.9 (2.2)	2.1 (2.2)	0.06
Median (IQR) gestation at birth (weeks)	39.0 (2.2) n = 60	39.3 (1.5) n = 43	0.43
Mean ± SD birth weight^e^ (grams)	3275.3 (±703.0) n = 60	3375.3 (±443.3) n = 43	0.41

HSC: humanitarian source countries, IQR: interquartile range, SD: standard deviation.

^e^Live births only.

Except where specified, n = 61 for the non-HSC group and 45 for the HSC group.

## Discussion

This study presents a comprehensive description of maternal health, pregnancy care attendance and pregnancy outcomes for 2173 women born in HSC and non-HSC from three African regions, to explore the impact of refugee background in addition to migrant background. General health appeared poorer and social disadvantage greater (including interpreter need) in women from HSC. Late first pregnancy care visits at the hospital were common in all groups. We present novel data suggesting specific HSC groups may be at greater risk of particular adverse pregnancy outcomes compared to non-HSC groups from similar world regions, such as BBA in the North African HSC group and GDM in the Middle and East African HSC group. Stillbirth was most common in the West African HSC group but was also more common than the national figures in most groups.

### Maternal general health

Compared to the non-HSC groups, poorer general health (underweight, infectious disease, Vitamin D deficiency), social disadvantage (interpreter need, teenage pregnancy, living in relatively socio-economic disadvantaged areas, no spouse) and FGM were generally more common in the HSC groups. Many of these are associated with adverse pregnancy outcomes [14,21-24]. There is a paucity of research literature regarding the general health of African migrant women and those of refugee background specifically [25,26]; these findings contribute to addressing this and indicate that African women of refugee background have greater health and wellbeing needs in pregnancy.

### Pregnancy care attendance

Early pregnancy care provides an opportunity to improve maternal health, connect women with social support and monitor maternal risk factors for adverse pregnancy outcomes. Pregnancy care attendance and timing of first pregnancy care visit at the hospital did not differ between non-HSC and HSC groups within any region. However, late first pregnancy care visits were more common than nationally (22-35%) in each group [20] and more common in the North African and West African groups than in the general hospital population. This is consistent with previous research reporting late first pregnancy care to be more common among African women than resettlement country-born women [7]. Pregnancy care in the first trimester is recommended for all women [27] and late first pregnancy care has been associated with adverse pregnancy outcomes [22], including among African migrant women [7]. While the Birthing Outcomes System does not capture appointments with non-hospital service providers, given the greater risk of adverse pregnancy outcomes among migrant women from Africa, early hospital pregnancy care could be vital and published research exists to inform the development of culturally appropriate strategies to address barriers to accessing early pregnancy care [28-31].

### Pregnancy outcomes

While pregnancy outcomes were not universally poorer among women of refugee background, each HSC group appeared to have high incidences of specific adverse outcomes. Our findings are consistent with previous research comparing African-born women to resettlement country-born women and advance knowledge by indicating additional risk associated with refugee background, above that associated with migrant background. GDM was independently associated with HSC-birth among women from Middle and East African countries and was more common than previous reports for African populations in Australia (4-10%) [32,33]. In the USA, higher GDM incidence has also been reported among Middle and East African women compared to USA-born women [34,35]. Health professional awareness of this may facilitate appropriate screening; culturally appropriate GDM education and management would be expected to have benefits for both maternal and neonatal health, especially as the highest incidence of admission to special or intensive neonatal care units was observed in this group (24%).

Performing an episiotomy in women who have experienced FGM is sometimes used as a strategy to reduce the risk of severe perineal trauma [14,36,37]. In our data women from Sudan (the only country in the North African HSC group) had significantly fewer episiotomies than women from North African HSCs. While not reaching statistical significance, serious perineal trauma appeared more common in the Sudanese group suggesting a possible need for this preventive practice; however further investigation is needed. Contributors to severe perineal trauma in these populations are not fully understood but are likely to include FGM severity which varies by country [14]. While FGM severity was not available for this study, the most severe form (involving infibulation) is the most common form in women from Sudan [14].

Women from Sudan were less likely to have non-cephalic presentation, caesarean section or assisted vaginal birth than the North African non-HSC group. Further investigation revealed that a larger proportion of women from Sudan had emergency caesarean sections and a smaller proportion had repeat caesarean sections. This is consistent with research in which a mostly Sudanese group of women in Australia was less likely to have elective caesarean sections than the hospital population [29]. Caesarean sections were less common among women from West African HSC than in the West African non-HSC group, reflecting a high caesarean section incidence in the non-HSC group. Past research regarding maternal origin and caesarean section has largely considered Africa as a single region [29,38] or combined Middle, East and West African regions [12]. Most research has noted a higher incidence of caesarean section among African, East African and Somali migrants, compared to resettlement country-born women [6,11,12,39]. A study that investigated migration status found little difference in incidence of caesarean section between African women of refugee and non-refugee background [38]. Authors of previous research have suggested the need for further region-based comparisons in this area [12] and our findings support and contribute to this by indicating differences between women of refugee and non-refugee background from specific African regions.

African migrant women have previously been reported to be less likely to use analgesia during labour than resettlement country-born women [6,29,40]. Our study builds on this by observing that women from Middle and East African HSC were less likely to use analgesia in labour than women from non-HSC, perhaps reflecting cultural perceptions of childbirth [41] or perhaps communication barriers with the greater need for interpreters. A smaller proportion of Sudanese women had induced labour and this may have contributed to the lower proportion of analgesia use in this group.

BBA was most common among women from Sudan with an incidence considerably higher than nationally (0.5%) [20]. BBA has been associated with neonatal morbidity and mortality [42,43], and postpartum haemorrhage [43,44]. Therefore, Sudanese-focused strategies encouraging engagement with pregnancy care are indicated.

There was evidence of poor neonatal outcomes in both HSC and non-HSC groups. Stillbirth incidence did not significantly differ within any region but was higher than the national rate (0.7%) [20] in five of the six groups, consistent with previous reports that women of African origin have a greater risk of stillbirth than resettlement country-born women [6-8]. The stillbirth incidence in the West African HSC group (4%) is especially concerning. Additionally, the West African non-HSC group had high incidences of preterm birth, low birth weight and admission to special or intensive neonatal care units, compared to national data [20]. This is consistent with previous studies noting adverse neonatal outcomes in women from West, Middle and East Africa combined, compared to resettlement country-born women [12,45]. The West African population size in our study was small, limiting the conclusions that can be drawn; however, little research has focussed specifically on migrants from West Africa and these results indicate further descriptions of pregnancy outcomes in this population are needed.

### Strengths and limitations

This study has limitations including that potential cultural and language barriers may contribute to over- or under-representation among wellbeing factors reliant on self-report e.g. spousal relationships. Some variables were only routinely collected from partway through the study time period (e.g. BMI); consequently sample size was reduced for these variables. Country of birth was used as a proxy for refugee background, however, in accordance with our previously published work [17], humanitarian and non-humanitarian source countries were rigorously selected to limit misclassification of individuals as much as possible. Year of arrival in Australia would have complemented country of birth when selecting probable refugee groups and is currently being incorporated into the Birthing Outcomes System. Despite this study having relatively large population sizes for a single maternity service in a resettlement country, due to the rare nature of most adverse pregnancy outcomes it was necessary to combine countries of birth into world regions in order to conduct statistical analysis. World region and country boundaries can be both dynamic and arbitrary, unavoidably heterogeneous ethnicities will have been grouped together. Identification of specific needs among each group of women belonging to a country or region would be ideal. Additionally, in the North African region, the population selection method resulted in a potentially more homogenous HSC group (Sudan only) being compared to a more heterogeneous non-HSC group (comprised of several countries of birth).

This study also has a number of strengths. It employed a rigorous, objective method to select current HSC and made the unique comparison to probable economic migrants from the same world regions. It described pregnancy outcomes for several African regions at a single maternity service, including West Africa which has not previously been described in detail. The large sample size enabled multivariable analysis to control for some potential confounders.

## Conclusions

In this unique study from one of the largest maternity services in Australia comparing women from three African regions, we have advanced knowledge on maternal health, pregnancy care attendance and pregnancy outcomes. It highlights the need for engagement with communities to improve early pregnancy care attendance as, within each African region, women born in HSC may be at greater risk of specific adverse pregnancy outcomes compared to women born in non-HSC. Early pregnancy care may also be especially important for women from West African non-HSCs who may have high rates of caesarean section and adverse neonatal outcome, although confirmatory research is needed. Awareness of these differing health needs would assist provision of appropriate care to improve the health of African women and their babies.

## Abbreviations

HSC: Humanitarian source country
FGM: Female genital mutilation
IRSD: Index of relative socioeconomic disadvantage
BMI: Body mass index
SD: Standard deviation
IQR: Interquartile range
CI: Confidence interval
HIV: Human immunodeficiency virus
GDM: Gestational diabetes mellitus
BBA: Unplanned birth before arrival at hospital
